# Antibiotika und Phagen: Auswirkungen einer kombinierten Therapie auf Empfindlichkeit und Resistenz bakterieller Infektionserreger

**DOI:** 10.1007/s00103-026-04232-8

**Published:** 2026-04-13

**Authors:** Peter Heisig, Anke Heisig

**Affiliations:** Institut für Biochemie und Molekularbiologie, Universität Hamburg, Bundesstraße 45, 20146 Hamburg, Deutschland

**Keywords:** Antibiotikaresistenz, Bakteriophage, Endolysin, Adaption, Kombinationstherapie, Antibiotic resistance, Bacteriophage, Endolysin, Adaption, Combination therapy

## Abstract

Steigender Antibiotikaverbrauch, auch zu nichttherapeutischen Zwecken, bei gleichzeitig rückläufiger Entwicklung neuer Wirkstoffe hat zu einer globalen Zunahme antibiotikaresistenter Bakterien geführt. Vor dem Zeitalter der Antibiotika wurden bereits Bakteriophagen (Phagen) zunächst in Frankreich und Georgien, später auch in den USA und Russland eingesetzt. Heute finden sie zunehmend Anwendung bei der Behandlung von Infektionen durch antibiotikaresistente Bakterien – einzeln oder auch in Kombination mit Antibiotika.

Eine Kombination von Phagen mit Antibiotika reduziert die Wahrscheinlichkeit einer gleichzeitigen Resistenzentwicklung gegen beide Wirkstoffe. Im Gegensatz zu chemisch definierten Antibiotika besitzen Phagen die Fähigkeit, sich durch genetische Mechanismen an resistente Wirtszellen anzupassen. Jedoch könnten bakterielle Mutationen, die Antibiotikaresistenz vermitteln, gleichzeitig die Wirksamkeit von Phagen einschränken. Zur Vermeidung solcher Effekte sind detaillierte Kenntnisse der Wirk- und Resistenzmechanismen des Antibiotikums sowie der Vermehrungsstrategie des Phagen erforderlich. Für die einzelnen Schritte im Vermehrungszyklus lytischer Phagen werden die für die jeweils mit Antibiotikaresistenz assoziierten Wirtzellfaktoren möglichen Auswirkungen auf Resistenz gegen entsprechende Phagen analysiert. Anhand ausgewählter Kombinationen zeigt sich, dass Resistenz gegen eine Komponente häufig mit erhöhter Empfindlichkeit gegenüber der anderen einhergeht, was die Wahrscheinlichkeit für eine gleichzeitige Resistenz gegen beide deutlich reduziert. Besonders effizient erscheinen synergistische Kombinationen, etwa aus Antibiotikum und Endolysin, einem bakterizid wirkenden Phagenenzym.

## Abstract (en)

The increasing use of antibiotics both in human therapy and for non-therapeutic applications with simultaneous reduction in antibiotic drug development have led to a world-wide rise in antibiotic resistance. Before the age of antibiotics, bacteriophages (phages) were already used initially in France and Georgia and later in the USA and Russia. Today, phages are increasingly being used to treat infections caused by antibiotic-resistant bacteria either alone or in combination with antibiotics.

Combining phages with antibiotics reduces the likelihood of simultaneous resistance development against both agents. In contrast to chemically defined antibiotics, phages have the ability to adapt to resistant host cells through genetic mechanisms. However, bacterial mutants that confer antibiotic resistance could simultaneously limit the effectiveness of phages. To avoid such effects, detailed knowledge of the mechanism of action and resistance of the antibiotic as well as the replication strategy of the phage is required. For the individual steps in the replication cycle of the lytic phages, the possible effects on resistance to the corresponding phages are analyzed for host cell factors associated with antibiotic resistance. Based on selected combinations, it becomes evident that resistance to one component is often accompanied by increased sensitivity to the other, which significantly reduces the likelihood of simultaneous resistance to both. Particularly efficient appear to be synergistic combinations, such as those consisting of an antibiotic and endolysin, a bactericidal phage enzyme.

## Einleitung

Die zunehmende Zahl von Infektionen durch antibiotikaresistente Bakterien stellt eines der größten globalen Gesundheitsprobleme dar. Für das Jahr 2050 wird prognostiziert, dass Infektionen durch solche Erreger mit etwa 10 Mio. Todesfällen pro Jahr die häufigste Todesursache sein werden [1]. Neben der Zunahme multiresistenter (multi-drug resistant, MDR) und panresistenter (pan-drug resistant, PDR) Bakterien trägt insbesondere die seit Jahren rückläufige Entwicklung neuer, gegen resistente Erreger wirksamer Antibiotika wesentlich zur weiteren Resistenzentwicklung bei. Dabei stellt die Fähigkeit von Bakterien, sich durch genetische Veränderungen rasch an Antibiotika anzupassen, einen entscheidenden Vorteil dar. Dagegen dauert die Weitentwicklung „klassischer“, an die jeweilige Resistenzsituation angepasster Antibiotika mittels chemischer Synthese bis zur Zulassung durchschnittlich etwa 15 Jahre [2].

Ein bereits seit über hundert Jahren bekannter biologischer Ansatz zur Bekämpfung verschiedenster bakterieller Erreger ist die Behandlung mit Phagen, die nach Erfolgen mit der Behandlung bakterieller Wundinfektionen bei französischen Soldaten im ersten Weltkrieg eingesetzt wurden. Der sensationelle Erfolg von Penicillin in den 1940er-Jahren und die Weiterentwicklung von Antibiotika, verdrängte den therapeutischen Einsatz von Phagen. Sie kamen nur noch in einigen osteuropäischen Ländern, zunächst vor allem in Georgien und Russland zum Einsatz, später auch in den USA. Seit einigen Jahren wird dieser Ansatz als vielversprechende Alternative in einigen europäischen Ländern, wie Polen, Belgien sowie in Deutschland im Rahmen von Einzelfallbehandlungen von Infektionen mit multiresistenten Erregern eingesetzt [3]. Phagen sind Viren, die den Vorteil besitzen, dass sie ausschließlich Bakterien infizieren und abtöten können. Ein weiterer Vorteil besteht darin, dass sie nur bestimmte Stämme ausschließlich bakterieller Spezies infizieren, wodurch das bestehende Mikrobiom des Patienten meist nicht beeinträchtigt wird. Darüber hinaus weist eine Therapie mit Phagen eine sehr gute Verträglichkeit bei diversen Applikationswegen auf. Der wichtigste Vorteil liegt in ihrer Fähigkeit, sich während der Therapie schnell an bakterielle Mutanten mit verringerter Empfindlichkeit gegenüber dem ursprünglich eingesetzten Phagen anzupassen.

Bei der beschriebenen Mikroevolution kommt es allerdings aufgrund der gegenseitigen Abhängigkeit zwischen Phagen und ihrer Wirtszelle (bakterieller Erreger) ohne weitere Wirtsfaktoren (Immunreaktion) nicht zu deren vollständiger Elimination. Deshalb wird häufig eine Kombination von Phagen mit Antibiotika eingesetzt. Bei dieser Therapie ist allerdings nicht immer vorher bekannt, ob dabei synergistische oder antagonistische Interaktionen zwischen Antibiotikum und Phagen bestehen.

Für eine therapeutische Anwendung von Phagen kommen folgende Ansätze in Betracht:

1. Die direkte enzymatische Lyse der Zellwand einer Wirtszelle durch Lysine einzelner Phagen oder eines Cocktails aus mehreren Phagen ohne Zusatz eines Antibiotikums [4].
2. Die Lyse der Wirtszelle durch eine antibakteriell wirkende Kombination aus Phage und Antibiotikum [4].
3. Die Reduktion lokaler protektiver Mechanismen von Wirtszellen, z. B. durch enzymatischen Abbau von Biofilmen durch Phagen, um den Zugang von Antibiotika zu ihren jeweiligen Angriffspunkten zu erleichtern und damit deren Aktivität zu steigern [5]. Ein anderes Beispiel hierfür ist die Desintegration der Membranen von Wirtszellen [6].
4. Der Einsatz von rekombinanten Phagen, die Gene für bestimmte Wirtsfaktoren tragen und in der Wirtszelle exprimieren können, wodurch z. B. eine Aktivierung endogener autolytischer Prozesse bewirkt wird ([4, 7]; Infobox 1).

Ziel dieses Beitrags ist es, an ausgewählten Beispielen den gegenseitigen Einfluss von Antibiotika bzw. Phagen auf die jeweilige Empfindlichkeit bakterieller Infektionserreger vorzustellen und, soweit bekannt, zugrunde liegende Mechanismen zu erläutern. Im Kontext einer Kombinationstherapie werden für die einzelnen Schritte im Vermehrungszyklus lytischer Phagen die Wirk- und Resistenzmechanismen derjenigen Antibiotika beschrieben, die an den beteiligten Wirtszellstrukturen angreifen (siehe auch Infobox 1).

## Interaktionen zwischen bakteriellen Wirtszellen und Phagen

Phagen, als die weltweit häufigsten biologischen Einheiten, bestehen aus Nukleinsäure (DNA oder RNA jeweils einzelsträngig, ss = „single-stranded“, oder doppelsträngig, ds = „double-stranded“), die von einer Hülle aus (Glyco‑)Proteinen umgeben ist, teilweise auch in Kombination mit Polysacchariden. Typischerweise verfügen Phagen über Anhängsel in Form von Proteinfilamenten, mit denen sie sich an eine als Rezeptor bezeichnete Oberflächenstruktur der Wirtszelle anheften. Für die Klassifizierung und verwandtschaftliche Zuordnung von Phagen wurden zunächst diese Bestandteile als Merkmale verwendet. Eine Einordnung der verschiedenen Phagen in einen monophyletischen Stammbaum, der auf einer mehr oder weniger ausgeprägten Homologie von Genomsequenzen beruht, ist aufgrund des Fehlens eines „Urphagen“, von dem sich alle existierenden Phagen ableiten, nicht möglich. Ebenso existiert kein bei allen Phagen vorkommendes Gen, vergleichbar dem 16S-rRNA-Gen bei Bakterien, das für eine auf DNA-Sequenzhomologie basierende phylogenetische Einteilung geeignet wäre [8]. Eine aktuell überarbeitete Taxonomie nutzt eine Kombination aus genomweiter Sequenzhomologie und einzigartigen funktionellen Proteinen [9].

Phagen sind zwingend auf bakterielle Wirtszellen angewiesen, deren enzymatische Ausstattung sie zur Replikation ihrer Nukleinsäure nutzen sowie zur Produktion der Moleküle, die für die Herstellung vollständiger infektiöser Phagenpartikel erforderlich sind. Nach Abschluss der verschiedenen Schritte eines Vermehrungszyklus in der Wirtszelle wird die dabei gebildete neue Phagenpopulation für den nächsten Vermehrungszyklus freigesetzt (lytischer Weg). Dafür wird die Zellwand durch Phagen-codierte lytische Enzyme aufgelöst, wie z. B. Endolysin, Holin, Spanin, die z. T. auch in isolierter Form therapeutisch eingesetzt werden [10]. Findet die Phagenvermehrung erst nach zwischenzeitlichem Einbau der Phagen-DNA in das Wirtszellchromosom statt, handelt es sich um einen lysogenen Vermehrungszyklus [11].

Die dabei ablaufenden wesentlichen Interaktionen zwischen Bakteriophage und Wirtszelle umfassen:

1. *Adsorption*: Bindung des Phagen an die Wirtszelle über molekulare Interaktion zwischen einer Teilstruktur der Phagenoberfläche als Ligand und einem Phagenrezeptor in der äußeren Wirtszellhülle. Dabei können Polysaccharidstrukturen wie Einzelzelloberflächen-assoziierte Kapseln oder von Mikrokolonien sezernierte Biofilme als Barrieren wirken.
2. *Penetration:* Durchdringen der Zellhülle durch den Phagen, meist unter Beteiligung enzymatischer Aktivitäten mit intrazellulärer Freisetzung der Phagen-Nukleinsäure.
3. *Intrazelluläre Phase*: Nutzung der Wirtszellmaschinerie für 2 alternative Entwicklungswege:
  1. *Lytischer Zyklus:* Expression von Phagen-spezifischen Genen für Enzyme zur Replikation (z. B. bei RNA-Phagen für die Umschreibung in DNA) sowie gegebenenfalls zur Regulation der Transkription/Translation und zur Lyse der Wirtszelle für die Freisetzung neugebildeter Phagenpartikel bei der lytischen Vermehrung.
  2. *Lysogener Zyklus:* Integration von Phagen-DNA in das Wirtszellgenom, wofür sowohl Wirtszell-codierte, als auch einzelne Phagen-codierte Enzyme benötigt werden. Die integrierte Phagen-DNA wird im Rahmen der Replikation der Wirtszell-DNA vermehrt (lysogener Weg).
4. *Induktion*: Exzision der integrierten Phagen-DNA, z. B. durch Auslösen von Antibiotikastress durch Fluorchinolone unter Fortsetzung der Reaktionen (s. 3.1.)

An diesen Schritten können z. T. auch Reaktionen von bakteriellen Zielstrukturen für Antibiotika beteiligt sein, was Einfluss auf eine antibakterielle Wirkung von Phagen haben kann ([12]; Infobox 2 beschreibt Prinzipien und Wechselwirkungen bei kombinierter Therapie mit Phagen und Antibiotika).

## Wechselwirkungen zwischen Phagen und Antibiotika

### Wechselwirkungen bei der Adsorption an die Wirtszelle

#### Grampositive Wirtszelle.

Für die Adsorption können Phagen verschiedene extern zugängliche Strukturen auf der Wirtszelloberfläche nutzen. Bei grampositiven Bakterien sind das z. B. Zellwand-assoziierte Teichonsäuren (wall teichoic acids, WTA), Cytoplasmamembran-assoziierte Teichonsäuren (lipoteichoic acids, LTA), Peptidoglycan und Flagellen [13].

Von besonderem Interesse für verbesserte therapeutische Ansätze durch Anwendung von Phagen – auch in Kombination mit Antibiotika – ist Peptidoglycan. Es dient zum einen als ein Rezeptor für Phagen bei der Adsorption an Zellen, z. B. von *Listeria monocytogenes *[14]. Zum anderen ist das Peptidoglycan auch eine Zielstruktur für Phagen-Endolysine, die erst im letzten Schritt der Phagenvermehrung im Cytoplasma der Zelle gebildet werden und die Lyse des Peptidoglycans katalysieren, um die neu gebildeten Phagen freizusetzen. Bei einigen Phagen ist ein Phagen-Lysin aber auch extern mit der Spitze der Schwanzproteine aus der Phagenhülle kovalent verbunden. So wirkt es bereits bei der Adsorption, dem ersten Kontakt eines Phagen-Schwanzproteins mit der Außenseite der Wirtszelle. Das Phagen-Lysin wird dabei aktiviert und hydrolysiert lokal die Peptidoglycanschicht. So wird die Adsorption des Bakteriophagen an einen Phagenrezeptor in der Cytoplasmamembran und darüber der Eintritt des Phagen in die Wirtszelle erleichtert [15]. Ein Beispiel für eine therapeutische Nutzung eines solchen Enzyms ist die Peptidoglycanhydrolase Exebacase, ein rekombinant hergestelltes Phagen-Lysin mit schneller lytischer Wirkung gegenüber *S.aureus-*Isolaten [16].

Methicillin-resistente *Staphylococcus-aureus-*(MRSA-)Stämme zeigen häufig eine Resistenz gegenüber nahezu allen β‑Lactam-Antibiotika, die auf der Kombination aus β‑Lactamase-Bildung (*blaZ*) und der Expression eines vermindert empfindlichen Penicillin-Bindeproteins PBP2′ (*mecA-*Genprodukt) beruht. Bei Behandlung solcher Stämme mit Phagen, die speziell MRSA-Stämme infizieren, konnten Mutanten selektiert werden, bei denen die Empfindlichkeit für β‑Lactam-Antibiotika wiederhergestellt war. Eine vollständige Genomanalyse ergab keine Mutationen in *blaZ *oder *mecA*, jedoch fanden sich Mutationen, die in 2 Fällen Auswirkungen auf die Zellwandsynthese haben: In einem Fall (*femA*-Gen) war die Quervernetzung der Peptidoglycanstränge reduziert und im anderen Fall (*spoVG*-Gen) wurde die Kontrolle über den Zellwandabbau aufgehoben. Durch diese Schwächung der Zellwand-stabilisierenden Peptidoglycanschicht als Antwort auf die Phageninfektion waren die nun Phagen-resistenten Zellen wieder empfindlich für β‑Lactam-Antibiotika [17].

Daptomycin ist ein weiteres, nur gegen grampositive Bakterien wirksames Antibiotikum, das vor allem bei Infektionen durch MRSA-Stämme eingesetzt wird. Nach Durchtritt durch die mehrschichtige Peptidoglycanschicht kann Daptomycin die Cytoplasmamembran desintegrieren, was zum Zelltod durch osmotische Lyse führt. Aufgrund zunehmend zu beobachtender Daptomycin-Resistenz unter der Therapie wurden Daptomycin-empfindliche Stämme und deren direkte resistente Nachkommen darauf untersucht, welchen Effekt eine Kombination von Bakteriophagen mit Daptomycin zeigt. Es wurde eine erhöhte Wirksamkeit der Phagen beobachtet [18].

#### Gramnegative Wirtszelle.

Bei gramnegativen Erregern dienen neben Pili und Flagellen auch Komponenten der äußeren Membran als Phagenrezeptoren. Dazu zählen Porine, die entweder einzeln den Influx von Nährstoffen vermitteln oder als Bestandteil von Effluxpumpen am Export von Substanzen wie Antibiotika beteiligt sind, sowie Lipopolysaccharide bzw. deren Bestandteile wie Lipid A. Einige dieser Strukturen spielen zugleich eine zentrale Rolle bei der Ausbildung von Resistenzen gegenüber verschiedenen Antibiotika.

So dienen Porine wie OmpC und OmpF bei *Escherichia coli* als Transmembranporen der Aufnahme von hydrophilen Nährstoffen, ermöglichen jedoch zugleich den Eintritt strukturell unterschiedlicher Antibiotika, darunter Chloramphenicol, Tetracyclin, Azithromycin und Fluorchinolone. Mutationen, die die Expression der *ompC/ompF*-Gene reduzieren, führen daher zu einer verminderten Empfindlichkeit gegenüber diesen Antibiotika [19]. Für Phagen, die diese Porine zur Adsorption nutzen, ist damit aber auch die Zahl der Andockstellen reduziert. Infolgedessen ist der Zugang für solche Phagen eingeschränkt.

Der Bakteriophage TLS nutzt zur Wirtszelladsorption ein anderes Porin, nämlich TolC [20]. Dieses ist Bestandteil der wichtigsten unspezifischen MDR Effluxpumpe AcrAB-TolC bei *E. coli*, deren Expression ebenso wie die von *ompC* und *ompF* unter der Kontrolle des *mar*-Operons („**m**ultiple **a**ntibiotic **r**esistance“) steht. Bei erhöhter Konzentration verschiedener Antibiotika (s. oben) gelangen zunächst Antibiotika durch die Porine OmpC/OmpF in die Zelle. Das führt zur Aktivierung des *mar*-Operons mit der Folge, dass gleichzeitig die Expression der Gene für die Effluxpumpe AcrAB-TolC erhöht, während die für OmpC/OmpF reduziert wird [21]. Die Zellen erlangen so eine Resistenz gegen solche Antibiotika, die OmpF/OmpC und AcrAB-TolC nutzen. Durch Selektion mit Fluorchinolonen lassen sich *mar*-Mutanten isolieren. Die entsprechenden *mar*-Mutationen betreffen vor allem das *marR*-Gen für den Repressor des *mar*-Operons und bewirken eine erhöhte Expression der Effluxpumpe AcrAB-TolC sowie eine Reduktion des Porins OmpF und erhöhen damit die Fluorchinolonresistenz. Werden Mutanten selektiert, die gegen den Phagen TLS resistent sind, so finden sich Mutationen in *tolC*, welche die Bindungsaffinität von TLS reduzieren, aber nicht die Effluxaktivität von TolC im Komplex mit AcrAB beeinträchtigen [20].

Mit einem Stamm von *Pseudomonas aeruginosa*, der aufgrund erhöhter Aktivität der MDR Effluxpumpen MexAB-OprM und MexXY-OprM (analog zu AcrAB-TolC bei *E. coli*) eine verminderte Empfindlichkeit gegenüber mehreren Antibiotika aufweist, wurde eine Selektion mit dem lytischen Bakteriophagen OMKO1 durchgeführt [22]. Dieser nutzt das Porin OprM zur Adsorption. Eine Selektion mit OMKO1 führt zu resistenten Mutanten der Wirtszellen mit einer reduzierten Aktivität der beiden Effluxpumpen. Damit geht eine erhöhte Empfindlichkeit für mehrere Antibiotika einher. Das Beispiel des OMKO1-Phagen zeigt, dass vorhandene Resistenzmechanismen gegen bestimmte Antibiotika durch Infektion mit geeigneten Bakteriophagen umgangen werden können. Dies eröffnet die Option für eine Kombinationstherapie mit Nutzung von Phagen für die Wiederherstellung der Antibiotikaempfindlichkeit ([22]; Infobox 3 beschreibt die molekularen Grundlagen der Wirkungsweise von Kombinationstherapien).

Neben Porinen spielt auch der Lipid-A-Anteil der Lipopolysaccharidschicht eine Rolle als Rezeptor für die Adsorption bestimmter Phagen. Weiterhin beruht die bakterizide Wirkung von Polymyxin-Antibiotika, wie Colistin, ebenfalls auf einer Interaktion des – positiv geladenen – Antibiotikums mit der – negativ geladenen – LPS-Oberfläche. Untersuchungen an Colistin-resistenten *Klebsiella pneumoniae* Isolaten zeigen, dass diese Resistenz auf Strukturänderungen in der negativ geladenen Lipopolysaccharidschicht der äußeren Membran beruht. Diese Änderungen betreffen die enzymatische Umwandlung negativer in positiv geladene Oberflächenladungen. Die positiv geladenen Colistinmoleküle werden dadurch von der Oberfläche der äußeren Membran abgestoßen. Bestimmte Phagen mit einer negativ geladenen Oberflächenstruktur können dagegen besser an die LPS-Komponenten der Colistin-resistenten Wirtszelle mit reduzierter negativer Oberflächenladung adsorbieren. Der Erwerb von Resistenz gegen Colistin geht also einher mit einer erhöhten Empfindlichkeit gegenüber bestimmten Phagen [23].

### Wechselwirkungen bei der Biosynthese von Phagen-DNA/‑RNA, ‑mRNA und -Proteinen

Nach der Adsorption eines Phagenpartikels an die Wirtszelloberfläche folgt die Injektion der linearen Phagen-Nukleinsäure in das Cytoplasma. Diese wird umgehend zu einer dsDNA umgeschrieben und dann zirkularisiert. Diese Form der DNA wird direkt zum einen mit Wirtszell- oder Phagen-eigenen Enzymen (DNA-Polymerase, Topoisomerase, Ligase) repliziert, zum anderen dient diese als Vorlage für die Expression von Genen für Phagenproteine durch zelluläre (Ribozym des Ribosoms) bzw. Phagen-spezifische Enzyme (RNA-Polymerase). Der lytische Vermehrungszyklus endet mit dem Zusammenfügen der gebildeten Phagenkomponenten – Nukleinsäure und Proteine – zu neuen Phagenpartikeln, die anschließend durch die Wirkung von Endolysinen zur Lyse der Wirtszelle und zur Freisetzung der Phagen führen (eine Übersicht findet sich in [24]).

Die meisten Antibiotikaklassen greifen in einen der genannten Prozesse, die im Cytoplasma der Wirtszelle ablaufen, ein: Fluorchinolone und Novobiocin in die DNA-Replikation, Rifampicin in die Transkription und z. B. Aminoglycoside, Chloramphenicol, Lincosamide, Makrolide, Tetracycline/Glycylcycline in die Translation. Mögliche Wechselwirkungen mit Bakteriophagen betreffen daher insbesondere diese Prozesse, die für die Vermehrung von Wirtszelle und Phagen gleichermaßen essenziell sind. Dabei ist aber zu berücksichtigen, dass Antibiotika, welche Transkription und Translation hemmen, mit Ausnahme der Aminoglycosid-Antibiotika bakteriostatisch wirken, also die Zelle und auch den Phagen lediglich in einem Ruhezustand halten. Dies schließt auch die Bildung der für die Phagenfreisetzung erforderlichen Endolysine mit ein. Liegt dagegen eine Resistenz gegenüber diesen Antibiotika vor, können sowohl zelluläre als auch Phagen-codierte Proteine für die Phagenvermehrung und -freisetzung produziert werden. In diesem Fall werden die Phagen nach Lyse der Wirtszelle freigesetzt [25]. Dies stellt eine mögliche allgemeine Konsequenz von Antibiotikaresistenz für die Empfindlichkeit der Wirtszelle gegenüber Phagen dar. Bei detaillierter Betrachtung verschiedener Konstellationen in Abhängigkeit von der genetischen Ausstattung von Wirtszelle und Phage finden sich unterschiedliche und nicht immer direkt vorhersagbare Konsequenzen von Antibiotikawirkung/-resistenz für die Phagenresistenz/-empfindlichkeit.

So verfügen z. B. verschiedene Stämme von *Pseudomonas aeruginosa* über ein Phagenabwehrsystem auf der Basis von CRISPR-Cas, das Phagen-spezifische DNA-Sequenzen in der Phagen-DNA abbaut. Bestimmte Phagen können jedoch direkt nach Eindringen der Phagen-DNA ein eigenes Abwehrsystem aus *A*nti-*CR*ISPR-(ACR-)Proteinen aktivieren, um ihre DNA vor dem Abbau zu schützen. Durch translationshemmende Antibiotika wird jedoch die Bildung der ACR-Proteine verhindert, sodass die Phagen wieder von dem vorhandenen CRISPR-System eliminiert werden können. Die Antibiotikum-empfindlichen Wirtszellen sind also Phagen-resistent. Antibiotikum-resistente Wirtszellen erlangen dagegen eine Empfindlichkeit für die Phagen, da diese ACR-Proteine bilden können [26].

Neben der Wechselwirkung zwischen Wirtszelle und Phage kann aber auch eine direkte Interaktion zwischen einem kleinen Molekül, wie einem Aminoglycosid-Antibiotikum (z. B. Apramycin), und Phage einen Einfluss auf dessen antibakterielle Wirkung haben, wie z. B. bei dem dsDNA-Phagen λ: Direkt nach der Injektion der linearen dsDNA in *E.coli* Wirtszellen wird die λ‑DNA zirkularisiert und in das Wirtsgenom integriert (lysogener Zustand). Ein solches Verhalten zeigt auch ein anderer, aus *Streptomyces*-Arten isolierter Wirkstoff aus der Gruppe der planaren tricyclischen Anthracycline. Dessen inhibitorische Wirkung auf den DNA-Zirkularisierungsschritt wird mit einer Bindung an die lineare Form der dsDNA erklärt. Für Apramycin ist allerdings eine solche Bindung nicht beschrieben. Ist die Wirtszelle jedoch aufgrund der Bildung eines Aminoglycosid-modifizierenden Enzyms resistent gegen Apramycin, wird die Zirkularisierung der Phagen-DNA nicht inhibiert, sodass sich die Phagen unter Lyse der Wirtszelle vermehren können. Auch in diesem Fall führt der Erwerb von Resistenz gegen ein Antibiotikum zur Sensibilisierung für einen Phagen [27].

Inhibitoren der DNA-Replikation, wie Fluorchinolone, hemmen Typ-II-Topoisomerasen, welche die kompakte, überspiralisierte („supercoiled“) Form zirkulärer dsDNA-Moleküle sowohl von Phagengenomen wie auch des bakteriellen Chromosoms aufrechterhalten. Dazu wird ein DNA-Bereich um die Topoisomerase, die als A_2_B_2_ Tetramer vorliegt, gewunden. Unter Ausbildung einer kovalenten Bindung zwischen jeweils einem Tyrosinrest im aktiven Zentrum der beiden A‑Untereinheiten und jeweils einem Phosphatrest am 5′-Ende des DNA-Moleküls wird ein DNA-Doppelstrangbruch eingeführt und fixiert. Dadurch wird die DNA-Replikation gestoppt und über die Aktivierung eines Stresssystems die DNA in Fragmente gespalten, wodurch der bakterizide Effekt ausgelöst wird [28]. Das im Chromosom der Wirtszelle integrierte Genom des Phagen λ wird im Verlauf der Stressreaktion an spezifischen DNA-Sequenzen wieder ausgeschnitten (s. oben). Dabei können zusätzlich Abschnitte der Wirtszell-DNA mit herausgeschnitten werden. Diese DNA-Moleküle werden jeweils zirkularisiert und dienen als Vorlage für die Expression der darauf codierten Phagengene mit Bildung entsprechender Proteine, u. a. auch solcher, die zur Bildung infektiöser Phagenpartikel verwendet werden. Bei diesem Vorgang können auch chromosomale Gene, die für Resistenz gegen verschiedene Antibiotika codieren, mit dem Phagengenom zusammen verbreitet werden (Transduktion; [29]). In Fluorchinolon-resistenten lysogenen Wirtszellen (mit integrierter Phagen-DNA) wird die Topoisomerase-Reaktion nicht gehemmt und das Stresssystem nicht induziert. Fluorchinolon-resistente Zellen sind somit gleichzeitig empfindlich für Phagen [30].

Einen unerwarteten Effekt zeigt das Antibiotikum Novobiocin, das ebenfalls Typ-II-Topoisomerasen hemmt, jedoch nicht bakterizid wirkt, da es kompetitiv ATP von seiner Bindungsstelle in der ATPase-Region der B‑Untereinheit verdrängt und diese dadurch hemmt. Als Folge wird der DNA-Spaltungsschritt der A‑Untereinheiten gar nicht ausgeführt, die DNA-Replikation bleibt in einem Zwischenschritt stehen, jedoch ohne das Stresssystem zu induzieren [31]. Aufgrund der strukturellen Ähnlichkeit von Novobiocin zu ATP bindet Novobiocin aber auch an andere ATP-Bindeproteine der Wirtszelle. Dazu gehört z. B. das LptB-Protein als Teil des Lpt-Transportkomplexes für Lipopolysaccharid-(LPS-)Moleküle. Diese sind die Hauptkomponente der äußeren Membran gramnegativer Bakterien, wie z. B. *E. coli*. Novobiocin stimuliert die Transportfunktion von LptB, sodass der LPS-Anteil in der äußeren Membran steigt. Dadurch wird für die bakterizide Wirkung des Antibiotikums Colistin eine geringere Konzentration zur Bindung an LPS benötigt, um Novobiocin-empfindliche Zellen über die Desintegration der äußeren Membran durch Colistin zu lysieren. Diese synergistische Wirkung von Novobiocin und Colistin ist an *Acinetobacter baumannii* Zellen gezeigt worden [32]. Für Phagen, die zur Infektion an LPS binden, ist bei Gabe von Novobiocin eine synergistische Interaktion gezeigt worden [33].

Die letzte Phase der lytischen Phagenvermehrung in der Wirtszelle umfasst die Lyse der Wirtszellen mittels Phagen-spezifischer Enzyme mit anschließender Freisetzung der Phagen. Dafür werden die im Cytoplasma gebildeten Enzyme durch die Cytoplasmamembran zur Peptidoglycanschicht transportiert, wo sie die Peptidoglycanstränge bzw. deren intermolekulare Peptidketten hydrolysieren und durch die Destabilisierung der Zellwand die osmotische Lyse der Wirtszelle verursachen.

Zur Umgehung dieser Problematik wurde als Alternative die Kombination wirksamer Antibiotika mit isolierten Phagen-Lysinen geprüft. Bei dieser Anwendung muss gewährleistet sein, dass die Wirkung von Endolysinen auch dann erfolgt, wenn diese extern zusammen mit einem Antibiotikum auf die Erregerzellen einwirken. Mit dem biotechnologisch hergestellten Endolysin Exebacase konnte in Kombination mit Antibiotika dreier unterschiedlicher Klassen (Daptomycin, Oxacillin und Linezolid) gezeigt werden, dass selbst bei konstanter subMHK (1/16 der minimalen Hemmkonzentration) über einen Zeitraum von 28 Tagen mit schrittweise steigenden Antibiotikakonzentrationen keine Antibiotika-resistenten Mutanten von *Staphylococcus aureus* selektiert werden. Dieser synergistische Effekt wird damit erklärt, dass die Wirkung von Exebacase sehr schnell und unabhängig von der Proteinbiosynthese erfolgen kann, also auch in Anwesenheit von Linezolid als Inhibitor der ribosomalen Proteinsynthese [16]. Dieser Synergismus konnte auch mit Kombinationen anderer extern applizierter Endolysine und Antibiotika für verschiedene grampositive Erreger gezeigt werden [34]. Bei der Anwendung extern applizierter Endolysine auf gramnegative Erreger stellt die für Endolysine schwer penetrierbare äußere Membran eine Barriere dar, die der Zelle einen Schutz ihrer darunterliegenden dünnen Peptidoglycanschicht verleiht. Doch durch die Verwendung von Membranvesikeln, wie sie sich beim Zellwachstum gramnegativer Zellen aus Stücken der äußeren Membran abschnüren und dabei auch kleinere Enzyme und Peptide aufnehmen können, eröffnet sich eine effektive Möglichkeit zur externen Anwendung von isolierten Endolysinen in Kombination mit Antibiotika [35].

## Fazit

Die Zahl Antibiotika-resistenter Bakterien nimmt trotz verschiedener Strategien, wie der Suche nach strukturell neuartigen Naturstoffen, chemisch-synthetischer bzw. kombinatorisch-biosynthetischer Modifikation bekannter Leitstrukturen sowie des Einsatzes von Wirkstoffkombinationen, weiterhin zu. Die hohe genetische Variabilität von Bakterien stellt dabei das Hauptproblem dar, da bereits unter der Therapie Mutanten mit Resistenz gegenüber den eingesetzten Therapeutika selektiert werden können.

Eine „biologische“ Alternative mit mindestens gleich hoher genetischer Variabilität und entsprechender Adaptionsfähigkeit unter Therapie stellen Bakteriophagen dar, die mit hoher Spezifität gezielt Erregerzellen abtöten können. Phagen zeigen (1) einen engen Wirtsbereich, der eine Erregerstamm-spezifische Auswahl erfordert, (2) aufgrund der wechselseitigen Abhängigkeit zwischen Phage und Wirtsbakterium den Ausschluss einer vollständigen Eliminierung des Erregers und (3) auch die Möglichkeit spontan auftretender Phagen-resistenter Bakterienmutanten. Durch Gabe einer Kombination aus Antibiotikum und Phage bzw. Phagengemisch werden diese unerwünschten Reaktionen weitestgehend ausgeschlossen.

Die aufgeführte Übersicht verschiedener Kombinationen aus Phage und Antibiotikum mit Angriffspunkt im Vermehrungszyklus des betreffenden Phagen zeigt für Mutanten mit Resistenz gegen das jeweils zur Selektion eingesetzte Agens (Antibiotikum bzw. Phage) in fast allen Fällen eine gleichzeitig erhöhte Empfindlichkeit für das andere Agens (Phage bzw. Antibiotikum). Erfolge aus entsprechenden *In-vivo*-Versuchen (Tierversuch, klinische Einzelfallbeschreibung) unterstützen diese Anwendungsoption.

Angesichts der sehr guten Verträglichkeit von Phagenpräparaten und einer in den letzten Jahren enorm angestiegenen Zahl verschiedener z. T. sehr gut charakterisierter Phagen ist zukünftig eine breite Anwendung für eine erfolgreiche antibakterielle Therapie mit anpassungsfähigen Phagen in Kombination mit Antibiotika in greifbare Nähe gerückt. Eine Herausforderung für den breiten Einsatz, auch im ambulanten Bereich, besteht weiterhin in der schnellen Identifizierung und Verfügbarkeit wirksamer, in applizierbarer Form vorliegender Phagen für die Kombination mit einem geeigneten Antibiotikum. Ebenso ist noch Forschungsbedarf vorhanden, um die Bedingungen für eine erfolgreiche Kombinationstherapie Phage-Antibiotikum zu präzisieren, z. B. im Hinblick auf Abstimmung pharmakokinetischer Eigenschaften (Applikationsreihenfolge).

### Infobox 1 Ursachen der Resistenzentwicklung gegen Antibiotika und therapeutische Strategien zur Umgehung

Resistenz ist ein bereits seit Beginn der therapeutischen Nutzung von Antibiotika bekanntes Problem. Eine wesentliche Ursache ist die hohe Mutationsrate bei gleichzeitig großen Zellzahlen bakterieller Populationen. Hinzu kommt der horizontale Gentransfer, sowohl intrazellulär (Rekombination, Transposition) als auch zwischen Zellen (Konjugation, Transformation und Transduktion mittels Phagen). Durch das schnelle Wachstum entstehen große Populationen mit zahlreichen zufälligen genetischen Veränderungen, darunter auch solche, die eine erhöhte Anpassung an Stress z. B. durch Antibiotika aufweisen. Bisherige therapeutische Strategien umfassen:

1. die chemisch-synthetische Anpassung der Struktur von Antibiotika an genetisch veränderte Zielstrukturen,
2. die Kombination zweier Antibiotika unterschiedlicher Klassen und
3. die Kombination eines Antibiotikums mit einem Hemmstoff des entsprechenden Resistenzmechanismus.

Diese Ansätze zeigten jedoch häufig nur begrenzte und zeitlich eingeschränkte Wirksamkeit.

Die Anwendung von Bakteriophagen (kurz Phagen) als bakterizid wirksames biologisches System stellt insofern eine Alternative dar, als dass das Wirkprinzip selbst die Fähigkeit zur genetischen Adaption einschließt. Phagen können sich bei Auftreten bakterieller Resistenz aufgrund ihrer hohen Mutationsrate rasch zu Varianten weiterentwickeln, die wiederum gegenüber resistenten Bakterien wirksam sind.

### Infobox 2 Koevolution von bakteriellem Erreger und Phage

Eine Phagentherapie beruht auf der Koevolution von bakteriellem Erreger und Phage, ohne dass einer dabei völlig eliminiert wird. Aufgrund der hohen Spezifität von Phagen für oft nur einzelne Stämme einer bakteriellen Erregerspezies erfordert eine erfolgreiche Therapie in der Regel den Einsatz eines „Cocktails“ aus verschiedenen Phagen, deren Wirtsspezifität für ein jeweiliges Patientenisolat vorab *in vitro* nachgewiesen sein muss. Alternativ kann ein als wirksam getesteter Phage mit einem Antibiotikum bekannter Wirksamkeit kombiniert werden.

Bei einer kombinierten Therapie aus Phagen und Antibiotika ist es entscheidend, zu verstehen, ob sich die Wirkmechanismen beider Komponenten synergistisch, antagonistisch oder indifferent verhalten. Abhängig von den Zielstrukturen der Antibiotika – wie Zellwand, Membrankomponenten, Enzyme – werden unterschiedliche Prozesse beeinflusst, die auch für die Phagenvermehrung relevant sind, darunter Stofftransport, DNA-Replikation, Transkription oder Translation. Diese Prozesse verlaufen sowohl im Vermehrungszyklus als auch im bakteriellen Zellzyklus in definierter Abfolge.

### Infobox 3 Molekulare Grundlagen einer Kombinationstherapie – am Beispiel von Adsorptionsstrategien

Experimentelle Ansätze und theoretische Betrachtungen weisen darauf hin, dass sich bei einer Antibiotikum-Phage-Kombination keine Mutanten mit klinisch relevanter Resistenz gegen beide Komponenten in angemessener Zeit isolieren lassen. Die verschiedenen Stadien im Vermehrungszyklus eines Phagen sind assoziiert mit Komponenten verschiedener zellulärer Prozesse der Bakterien. So betrifft die Adsorption eines Phagen an seinen bakteriellen Rezeptor als erster Schritt der Phagenvermehrung verschiedene Komponenten der Zellhülle, die an dem Transport von Antibiotika beteiligt sind. Änderungen der Menge bzw. der Struktur solcher Komponenten betreffen bei gramnegativen Erregern z. B. 3‑teilige Effluxpumpen aus Pumpe, Verbindungsprotein und Porin. Eine vermehrte Expression solcher Pumpen erniedrigt die intrazelluläre Antibiotikumkonzentration (Resistenz), erhöht aber gleichzeitig die Menge an Oberflächenrezeptoren (Effluxpumpe) für die Adsorption entsprechender Phagen (erhöhte Phagenempfindlichkeit).

In gleicher Weise führt eine veränderte Oberflächenladung im Lipid-A-Anteil der äußeren Membran zur Resistenz gegen Colistin, während Phagen mit erhöhter Affinität für die veränderte Oberflächenladung eine bessere Wirksamkeit zeigen. Analog ist die Zellwand (Peptidoglycanschicht) ein Rezeptor für bestimmte Phagen grampositiver Bakterien. Da die Wirkung der β‑Lactam-Antibiotika auf der Hemmung der Quervernetzung der Peptidoglycanstränge beruht, wird damit gleichzeitig der Zugang von Peptidoglycan-spezifischen Phagen erleichtert. Somit geht β‑Lactam-Resistenz einher mit erhöhter Empfindlichkeit für entsprechende Phagen.

Antibiotika, wie z. B. Chloramphenicol, Tetracyclin, Makrolide, hemmen die Proteinsynthese und unterbinden in Phagen-infizierten Zellen gleichzeitig die Synthese Phagen-spezifischer Proteine (Antibiotikawirksamkeit bei gleichzeitiger Phagenresistenz).

## References

[1] Wall S (2019) Prevention of antibiotic resistance—An epidemiological scoping review to identify research categories and knowledge gaps. Global Health Action 12:1756191 10.1080/16549716.2020.1756191 PMC7782542 32475304

[2] https://www.deutsche-apotheker-zeitung.de/news/artikel/2025/11/19/luecke-in-der-entwicklung-neuer-antimikrobieller-wirkstoffe-wird-groesser, Letzter Zugriff: 27.01.2026

[3] Rohde C (2024) Grundlagen zu Phagen und ihrer therapeutischen Anwendung. Bundesgesundheitsblatt 68(6):600–607 10.1007/s00103-025-04051-3 PMC12129868 40314735

[4] Luong T, Salabarria AC, Roach DR (2020) Phage therapy in the resistance era: where do we stand and where are we going? Clinical Therapeutics 42:1659–1680 32883528 10.1016/j.clinthera.2020.07.014

[5] Mayorga-Ramos A, Carrera-Pacheco SE, Barba-Ostria C, Guaman LP (2024) Bacteriophage-mediated approaches for biofilm control. Frontiers in Cellular Infection Microbiology 14:1428637 39435185 10.3389/fcimb.2024.1428637 PMC11491440

[6] Euler CW, Assaf R, Hernandez A et al (2023) PlyKp104, a novel phage lysin for the treatment of *Klebsiella pneumoniae, Pseudomonas aeruginosa*, and other Gram-negative ESKAPE pathogens. Antimicrobial Agents and Chemotherapy 67:e01519-22 37098944 10.1128/aac.01519-22 PMC10190635

[7] Hupfeld M, Trasanidou D, Ramazzini L et al (2018) A functional type II‑A CRISPR-Cas system from *Listeria* enables efficient genome editing of large non-integrating bacteriophage. Nucleic Acids Research 46:6920–6933 30053228 10.1093/nar/gky544 PMC6061871

[8] Dion MB, Oechslin F, Moineau S (2020) Phage diversity, genomics and phylogeny. Nature Reviews Microbiology 18:125–138 32015529 10.1038/s41579-019-0311-5

[9] Zhu X, Tang L, Wang Z et al (2024) A comparative analysis of phage classification methods in light of the recent ICTV taxonomic revisions. Virology 594:110016 38461619 10.1016/j.virol.2024.110016

[10] Cahill J, Young R (2019) Phage lysis: Multiple genes for multiple barriers. Advances in Virus Research 103:33–70 30635077 10.1016/bs.aivir.2018.09.003 PMC6733033

[11] Salmond GPC, Fineran PC (2015) A century of the phage: past, present, and future. Nature Reviews Microbiology 13:777–786 26548913 10.1038/nrmicro3564

[12] Altamirano FLG, Barr JJ (2019) Phage therapy in the post-antibiotic era. Clinical Microbiological Reviews 32:e00066-18

[13] Dicks LMT, Vermeulen W (2024) Bacteriophage–host interactions and the therapeutic potential of bacteriophages. Viruses 16:478 38543843 10.3390/v16030478 PMC10975011

[14] Wendlinger G, Loessner MJ, Scherer S (1996) Bacteriophage receptors on *Listeria monocytogenes* cells are the N‑acetylglucosamine and rhamnose substituents of teichoic acids or the peptidoglycan itself. Microbiology 142:985–992 8936325 10.1099/00221287-142-4-985

[15] Leprince A, Mahillon J (2023) Phage adsorption to Gram-positive bacteria. Viruses 15:196 36680236 10.3390/v15010196 PMC9863714

[16] Oh J, Warner M, Ambler JE, Schuch R (2023) The lysin Exebacase has a low propensity for resistance development in *Staphylococcus aureus* and suppresses the emergence of resistance to anti-staphylococcal antibiotics. Microbiology Spectrum 11:e5261-22 10.1128/spectrum.05261-22 PMC10100934 36862002

[17] Tran M, Hernandez Viera AJ, Tran PQ, Nilsen E, Tran L, Mo CY (2023) Bacteriophage infection drives loss of β‑lactam resistance in methicillin-resistant *Staphylococcus aureus*10.7554/eLife.102743.2 10.7554/eLife.102743 PMC12245174 40637714

[18] Madison CL, Steinert ASJ, Luedeke CE, Hajjafar N et al (2024) It takes two to tango: preserving daptomycin efficacy against daptomycin-resistant MRSA using daptomycin-phage co-therapy. Microbiology Spectrum 12:00679–24 10.1128/spectrum.00679-24 PMC11619598 39470283

[19] Luo KW, Li PL, Zhai YJ et al (2024) Upregulating of outer membrane porin gene *omp*C contributed to enhancement of azithromycin susceptibility in multi-drug resistant *Escherichia coli*. Microbiology Spectrum 12:03918–23 10.1128/spectrum.03918-23 PMC10986464 38441474

[20] German GJ, Misra R (2001) The TolC protein of *Escherichia coli* serves as cell-surface receptor for the newly characterized TLS bacteriophage. Journal of Molecular Biology 308:579–585 11350161 10.1006/jmbi.2001.4578

[21] Alekshun MN, Levy SB (1999) The *mar* regulon: multiple resistance to antibiotics and other toxic chemicals. Trends in Microbiology 7:410–413 10498949 10.1016/s0966-842x(99)01589-9

[22] Chan BK, Sistrom M, Wertz JE et al (2016) Phage selection restores antibiotic sensitivity in MDR *Pseudomonas aeruginosa*. Science report 6:26717 10.1038/srep26717 PMC4880932 27225966

[23] Hao G, Chen AI, Liu M et al (2019) Colistin resistance-mediated bacterial surface modification sensitizes phage infection. Antimicrobial Agents Chemotherapy 63:e1609-19 10.1128/AAC.01609-19 PMC6879231 31570405

[24] Salmond GPC, Finera PC (2015) A century of the phage: past, present and future. Nature Reviews Microbiology 13:777–786 26548913 10.1038/nrmicro3564

[25] Vashisth M, Yashveer S, Anand T et al (2022) Antibiotics targeting bacterial protein synthesis reduce the lytic activity of bacteriophages. Virus Research 321:198909 36057417 10.1016/j.virusres.2022.198909

[26] Pons BJ, Dimitriu T, Westra ER, van Houte S (2023) Antibiotics that affect translation can antagonize phage infectivity by interfering with the deployment of counter-defenses. Proceedings of the National Academy of Science 120:e2216084120 10.1073/pnas.2216084120 PMC9942909 36669116

[27] Kever L, Hardy A, Luthe T et al (2022) Aminoglycoside Antibiotics inhibit phage infection by blocking an early step of the infection cycle. mBio 113:00o783-22 10.1128/mbio.00783-22 PMC9239200 35506667

[28] Bush NG, Diez-Santos I, Abbott LR, Maxwell A (2020) Quinolones: mechanism, lethality and their contributions to antibiotic resistance. Molecules 25:05662 10.3390/molecules25235662 PMC7730664 33271787

[29] Haaber J, Penades JR, Ingmer H (2017) Transfer of antibiotic resistance in *Staphylococcus aureus*. Trends in Microbiology 25:893–905 28641931 10.1016/j.tim.2017.05.011

[30] Gavric D, Knezevic P (2025) Antibiotic-mediated prophage activation: mechanisms, consequences, and therapeutic consequences. International Journal of Antimicrobial Agents 2025 in press: 107655. 10.1016/j.ijantimicag.2025.107655. 10.1016/j.ijantimicag.2025.107655 41167349

[31] DeMarini DM, Lawrence BK (1992) Prophage induction by DNA topoisomerase II poisons and reactive-oxygen species: role of DNA breaks. Mutation Research 267:1–17 1373845 10.1016/0027-5107(92)90106-c

[32] Mandler MD, Raidin V, Lee J et al (2018) Novobiocin enhances polymyxin activity by stimulating lipopolysaccharid transport. Journal of the American Chemical Society 140:6749–6753 29746111 10.1021/jacs.8b02283 PMC5990483

[33] McGee LW, Barhoush Y, Shima R, Henessy M (2023) Phage-resistant mutations impact bacteria susceptibility to future phage infections and antibiotic response. Ecology and Evolution 13:e9712 36620417 10.1002/ece3.9712 PMC9817185

[34] Abdelrahman F, Easwaran M, Daramola OI et al (2021) Phage-encoded endolysins. Antibiotics 10:124 33525684 10.3390/antibiotics10020124 PMC7912344

[35] Beveridge TJ (1999) Structures of Gram-negative cell walls and their derived membrane vesicles. Journal of Bacteriology 181:4725–4733 10438737 10.1128/jb.181.16.4725-4733.1999 PMC93954

